# Seagrass ecophysiological performance under ocean warming and acidification

**DOI:** 10.1038/srep41443

**Published:** 2017-02-01

**Authors:** Tiago Repolho, Bernardo Duarte, Gisela Dionísio, José Ricardo Paula, Ana R. Lopes, Inês C. Rosa, Tiago F. Grilo, Isabel Caçador, Ricardo Calado, Rui Rosa

**Affiliations:** 1 MARE - Marine and Environmental Sciences Centre, Laboratório Marítimo da Guia, Faculdade de Ciências da Universidade de Lisboa, Avenida Nossa Senhora do Cabo 939, 2750-374 Cascais, Portugal; 2 MARE - Marine and Environmental Sciences Centre, Faculdade de Ciências da Universidade de Lisboa, Campo Grande, 1749-016 Lisboa, Portugal; 3 Departamento de Biologia & CESAM, Universidade de Aveiro, Campus Universitário de Santiago, 3810-193 Aveiro, Portugal

## Abstract

Seagrasses play an essential ecological role within coastal habitats and their worldwide population decline has been linked to different types of anthropogenic forces. We investigated, for the first time, the combined effects of future ocean warming and acidification on fundamental biological processes of *Zostera noltii*, including shoot density, leaf coloration, photophysiology (electron transport rate, ETR; maximum PSII quantum yield, F_v_/F_m_) and photosynthetic pigments. Shoot density was severely affected under warming conditions, with a concomitant increase in the frequency of brownish colored leaves (seagrass die-off). Warming was responsible for a significant decrease in ETR and F_v_/F_m_ (particularly under control pH conditions), while promoting the highest ETR variability (among experimental treatments). Warming also elicited a significant increase in pheophytin and carotenoid levels, alongside an increase in carotenoid/chlorophyll ratio and De-Epoxidation State (DES). Acidification significantly affected photosynthetic pigments content (antheraxanthin, *β*-carotene, violaxanthin and zeaxanthin), with a significant decrease being recorded under the warming scenario. No significant interaction between ocean acidification and warming was observed. Our findings suggest that future ocean warming will be a foremost determinant stressor influencing *Z. noltii* survival and physiological performance. Additionally, acidification conditions to occur in the future will be unable to counteract deleterious effects posed by ocean warming.

Atmospheric carbon dioxide (CO_2_) levels have stepwise risen from preindustrial levels of 280 μatm to ~390 μatm (present day), being expected to increase up to 420–940 μatm by 2100^1^. As carbon dioxide concentration increases in Earth’s atmosphere, CO_2_ exchange between atmosphere and sea surface layer will vary, with ocean CO_2_ uptake increasing and seawater chemistry changing, accordingly^2^. According to the Intergovernmental Panel on Climate Change (IPCC), average ocean pH is expected to decrease between 0.13–0.42 units by the end of the 21^st^ century, depending upon the different representative concentration pathways (RCP) IPCC scenarios^1^. As a consequence, changes in seawater chemistry and subsequent shift in the relative proportion of dissolved inorganic carbon species (DIC), will ultimately lead to a continuous process known as ocean acidification, with predictable cascading effects on marine ecosystems^2^. Concomitantly, and as a result of enhanced greenhouse effect, sea surface temperature (SST) has risen. Even though global SST trends will differ at a regional, seasonal and interannual scale, it is expectable (IPCC’s 8.5 RCP scenario) that mean SST will increase between 2.6 °C−4.8 °C until 2100^3^.

Future changes in ocean’s physical and chemical properties are expected to pose severe impairments to marine biota, by directly affecting organisms’ ability to regulate acid-base equilibrium, as well as their growth, survival, metabolism, physiology and reproduction^2^,^4^. Within marine coastal environments, seagrasses play an essential ecological key role as ecosystem builders providing food, shelter and crucial nursery habitats for a wide range of species^5^. Additionally, they act as “ecological service providers” by promoting sea bottom stabilization and nutrient cycling, acting as buffer/trophic transfer zones to sensitive/neighboring habitats (i.e. coral reefs) and as “environmental status beacons” of coastal ecosystems^5^,^6^. Their worldwide distribution and key ecological importance within coastal environments is such that seagrasses may represent, to more or less extent, the marine “counter part” of tropical rainforests, in terms of carbon cycle/storage potential as well as biodiversity promoters^7^,^8^. As a result of seagrass meadows exposure to a variety of both biotic and abiotic environmental stressors, these ecosystems are increasingly experiencing a global decline^9^,^10^. Most of these pressures result from anthropogenic forcing, such as dredging activities (hydrodynamic shifts), runoffs from urban and agriculture areas (e.g. household chemicals, metals, fertilizers/pesticides, animal waste), commercial/recreational human activities (e.g. fishing, boat groundings) and increasing climate change derived impacts^10^,^11^.

Within a climate change perspective, increasing temperature (and heat wave events) can cause detrimental changes upon seagrass growth, survival and distribution^12^,^13^. Additionally, temperature may also influence seagrass photosynthesis, nutrient uptake and act as a sexual reproduction trigger^10^,^14^,^15^. In fact, as temperature rises, seagrass photosynthesis increases up until a point where an optimal value is reached, followed by a subsequent rapid decline^16^. Concomitantly, an increase in photosynthesis is accompanied by a faster growth and consequent higher respiration rate, which in turn leads to a net primary productivity compromise status and a negative carbon balance^14^. Therefore, it is of particular concern to understand how ecologically important species (i.e. seagrasses) will survive, adapt or even geographically relocate, when faced upon an increasing warming habitat. Ocean acidification conditions have also proven to promote deleterious effects in marine organisms^2^. Opposingly, photosyntetic organisms (e.g. marine angiosperms) are expected to benefit from these changes, once aqueous CO_2_ is a crucial photosynthesis resource (primary carbon source) and as oceans become more acidic so will DIC species in seawater tend to increase. Although nearly 90% of total DIC in seawater is available as bicarbonate ions^17^, seagrasses tend to use this ionic carbon source, for photosynthesis, rather inefficiently, although further studies are needed as to confirm this assumption^18^,^19^,^20^. Nevertheless, it is important to highlight that environmental stressors can act synergistically and subsequent effects will be dictated as a result of species ability to cope with combined stress increase or an augmented antagonistic output towards stress tolerance^21^,^22^. Thus, understanding the combined effects between increasing temperature and rising CO_2_ levels is of utmost importance and should be carefully addressed in order to empirically predict potential cascading effects over the marine environment and associated biota.

The dwarf eelgrass *Zostera noltii* (Hornemann 1832) has a wide geographical distribution, covering several Atlantic coastal areas, from Norway to Mauritania, including the Mediterranean and adjacent seas^23^. Although not critically endangered as other seagrass species, there is an increasing concern towards the conservation of *Z. noltii* and its ecological status, mainly as a result of habitat degradation and decreasing trends at a population level^24^. As a foundation species of many marine coastal habitats, ecological and socio-economic importance, it is imperative to understand if *Z. noltii* will be able to cope with the scenarios predicted for the oceans of tomorrow.

The aim of our study is to understand, for the first time, the combined effects of ocean warming and acidification upon shoot density, leaf coloration, photophysiology (electron transport rate: ETR and maximum PSII quantum yield: F_v_/F_m_) and photosynthetic pigments of a pioneer temperate seagrass species (*Z. noltii*). As seagrass response towards the combined effects of ocean warming and acidification is still poorly understood, this study will be a major contribution as to improve our knowledge on potential cascading effects at a species and subsequently population and community levels.

## Results

### Shoot density and leaf coloration

**Figure 1:**
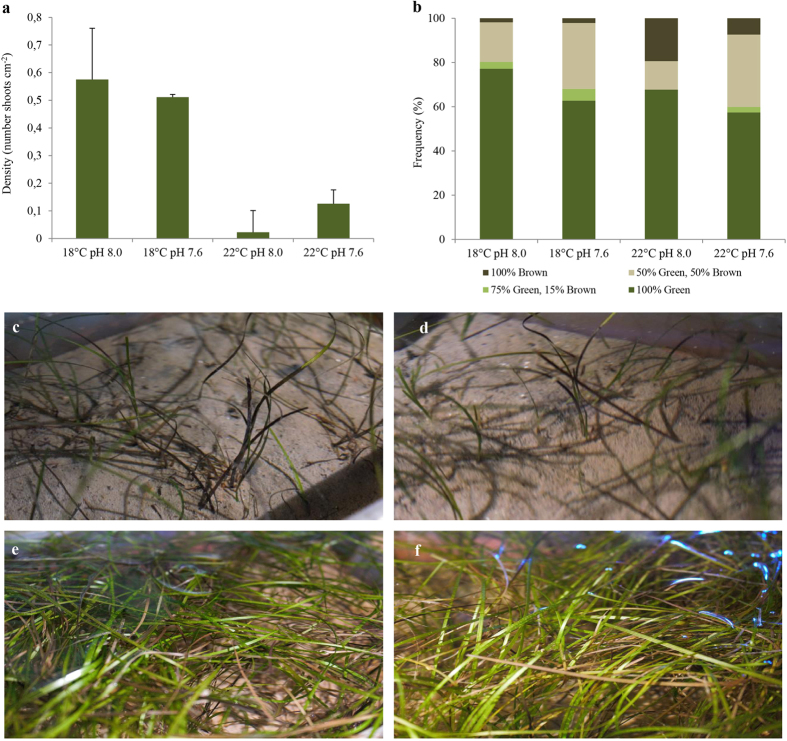
Impact of ocean warming (+4 °C) and acidification (ΔpH = 0.4) on *Zostera noltii*. (**a**) Shoot density (number of shoots cm^−2^) and (**b**) frequency of leaf colors per shoot (%). Exemplificative images of shoot density (T30), under the different experimental conditions [(**c**) 22 °C pH 8.0; (**d**) 22 °C pH 7.6; (**e**) 18 °C pH 8.0 and (**f**) 18 °C pH 7.6] are given. Values in panel (**a**) represent mean ± SD (i.e., n = 4 independent replicate systems, per experimental treatment).

At the beginning of the experimental treatments (T0), *Z. noltii* shoot density was 0.69 ± 0.16 shoots cm^−2^ (n = 16). Thirty days (T30) after exposure to the four scenarios investigated, *Z. noltii* shoot density was significantly reduced under warming conditions (Fig. 1; Supplementary Table S2). Shoot density varied from 0.58 shoots cm^−2^ under control conditions (18 °C, pH 8.0; Fig. 1a,e) to 0.02 shoots cm^−2^ under the warming and normocapnic (control pH) scenario (22 °C, pH 8.0; Fig. 1a,c). It is worth noting that hypercapnia induced a slight increase, although non-significant, in seagrass density under the warming scenario (Supplementary Table S2).

Regarding leaf coloration, seagrass kept under control conditions revealed a higher frequency of shoots with green leaves (77%; Fig. 1b), while seagrass exposed to the warming and normocapnic scenario displayed higher frequency of shoots with brown leaves (19%; Fig. 1b).

### Photobiology and pigments

**Figure 2:**
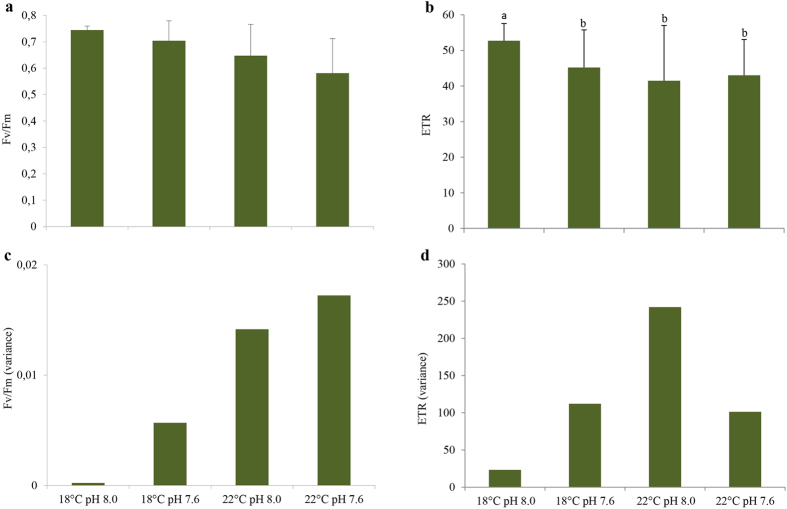
Impact of ocean warming (+4 °C) and acidification (ΔpH = 0.4) on *Zostera noltii*. (**a**) Maximum PSII quantum yield (F_v_/F_m_); (**b**) electron transport rate (ETR); (**c**) F_v_/F_m_ variance and (**d**) ETR variance among treatments. Values represent mean ± SD [(n = 40 per experimental treatment (i.e., n = 10 per replicate system (n = 4), per experimental treatment)]. Different letters represent statistical differences between temperature and pH treatments, following a significant interaction (p < 0.013).

Only temperature significantly affected the photophysiological parameters (ETR and F_v_/F_m_) of seagrass (two-way MANOVA: F_(2,151)_ = 25.96; p < 0.001; Pillai’s trace = 0.256) with no significant effects of pH or interaction between the explaining variables (two-way MANOVA: F_(2,151)_ = 2.76; p = 0.066; Pillai’s trace = 0.035; F_(2,151)_ = 1.60; p = 0.205; Pillai’s trace = 0.021, respectively). More specifically, warming caused a significant decrease in both F_v_/F_m_ and ETR levels, although it was not statistically significant in the case of ETR measured in seagrass exposed to acidification (Tukey *post-hoc* test: p < 0.013; Fig. 2a,b, respectively; Supplementary Table S2). It is worth noting that warming and hypercapnic treatment revealed the greatest F_v_/F_m_ variability (Fig. 2c), whereas the warming and normocapnic treatment showed the greatest ETR variability (Fig. 2d), among experimental treatments. A highly positive correlation between ETR variability and the frequency of shoots with 100% brown leaves was observed (r = 0.97; p < 0.05; Table 1).

**Figure 3:**
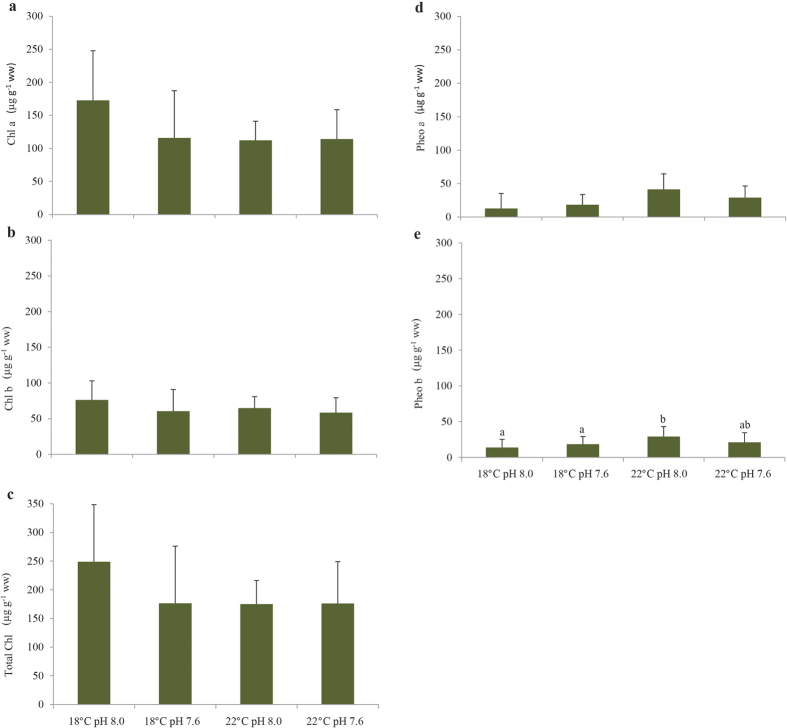
Impact of ocean warming (+4 °C) and acidification (ΔpH = 0.4) on *Zostera noltii*. (**a**) Chlorophyll *a*; (**b**) chlorophyll *b* ; (**c**) total chlorophyll; (**d**) pheophytin *a* and (**e**) pheophytin *b* contents (μg g^−1^ ww). Values represent mean ± SD [(n = 40 per experimental treatment (i.e., n = 10 per replicate system (n = 4), per experimental treatment)]. Different letters represent statistical differences between temperature and pH treatments, following a significant interaction (p < 0.013). Abbreviations: Chl *a*: chlorophyll *a*; Chl *b*: chlorophyll *b*; Total Chl: total chlorophyll; Pheo *a*: pheophytin *a*; Pheo *b*: pheophytin *b*; μg: microgram; g: gram; ww: wet weight.

**Figure 4:**
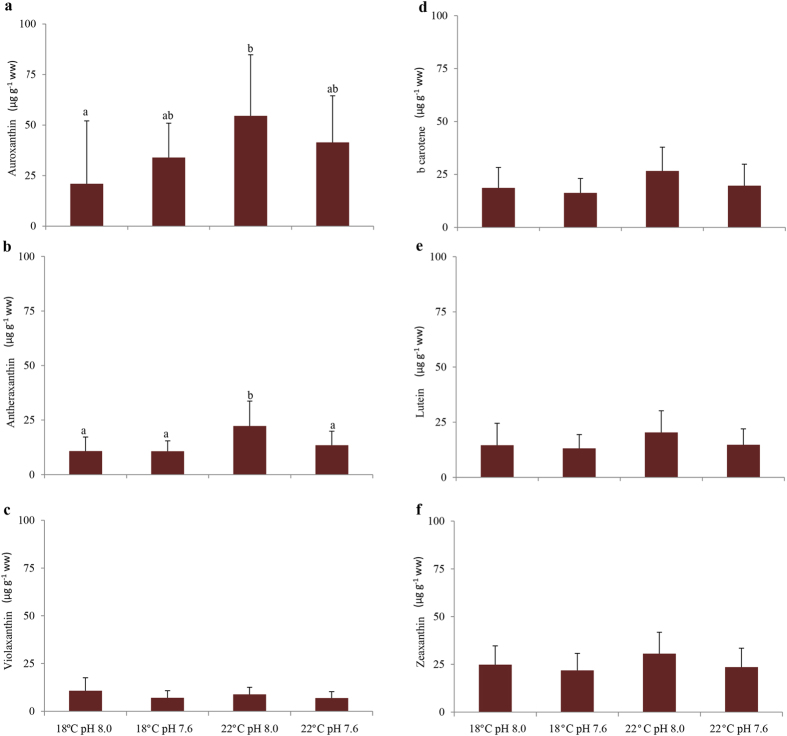
Impact of ocean warming (+4 °C) and acidification (ΔpH = 0.4) on *Zostera noltii*. (**a**) Auroxanthin; (**b**) antheraxanthin; (**c**) violaxanthin; (**d**) *β*-carotene; (**e**) luteín and (**f**) zeaxanthin contents (μg g^−1^ ww). Values represent mean ± SD [(n = 40 per experimental treatment (i.e., n = 10 per replicate system (n = 4), per experimental treatment)]. Different letters represent statistical differences between temperature and pH treatments, following a significant interaction (p < 0.013). Abbreviations: μg: microgram; g: gram; ww: wet weight.

Temperature and pH significantly affected photosynthetic pigment content (two-way MANOVA: F_(14, 65)_ = 2.44; p < 0.008; Pillai’s trace = 0.344; and F_(14, 65)_ = 2.01; p = 0.030; Pillai’s trace = 0.302, respectively) with a significant interaction between the two explaining variables (two-way MANOVA: F_(14, 65)_ = 1.99; p = 0.032; Pillai’s trace = 0.300). More specifically, chlorophyll *a* content was significantly lower under warming, whereas chlorophyll *b* content was influenced by both environmental factors (Fig. 3a,b; Supplementary Table S2). As for total chlorophyll content, it was not affected by either temperature or pH (Fig. 3c; Supplementary Table S2). It is worth noting that a highly positive correlation between chlorophyll b and the frequency of shoots with 100% green leaves was observed (r = 0.98; p < 0.05; Table 1). Pheophytin *a* and *b* showed an increasing trend with warming, under both normocapnic and hypercapnic conditions (Fig. 3d,e; Supplementary Table S2). Pheophytin *a* levels were highly negatively correlated with seagrass density (r = −0.96; p < 0.05; Table 1) and positively correlated with: i) frequency of shoots with 100% brown leaves (r = 0.96; p < 0.05; Table 1) and ii) ETR variability (r = 0.98; p < 0.05; Table 1). Identically, a significant positive correlation was recorded between these variables and pheophytin *b*, with the exception of seagrass density (p > 0.05; Table 1). Auroxanthin and antheraxanthin contents increased under warming and normocapnic condition (Fig. 4a,b), although it was only statistically significant for the latter accessory pigment (Supplementary Table S2). As for *β*-carotene, its content was significantly affected by both temperature and pH (Supplementary Table S2) with warming causing a significant increase in *β*-carotene, whereas acidification lead to a significant decrease in the content of this pigment (Fig. 4d). Regarding violaxanthin, only pH significantly affected the content of this pigment, similarly to what was observed regarding zeaxanthin content (Supplementary Table S2). In both cases, seagrasses exposed to hypercapnia showed a significant decrease in their pigments (Fig. 4c,f). Lutein did not significantly vary with both temperature and pH (Fig. 4e; Supplementary Table S2). Similar to pheophytin *a* and *b*, auroxanthin and antheraxanthin concentrations were positively correlated with ETR variability (r = 0.98 and 0.96, respectively; p < 0.05; Table 1). Moreover, while auroxanthin levels were positively correlated with pheophytin *a* and *b* (r = 0.98 and 0.99, respectively; p < 0.05; Table 1), antheraxanthin concentrations were also significantly correlated with the frequency of shoots with 100% brown leaves (r = 0.99; p < 0.05; Table 1). *β*-carotene levels were positively correlated with: i) antheraxanthin (r = 0,98; p < 0.05; Table 1), ii) lutein (r = 0,99; p < 0.05; Table 1), zeaxanthin (r = 0.97; p < 0.05; Table 1) and the frequency of shoots with 100% brown leaves (r = 0.97; p < 0.05; Table 1).

**Figure 5:**
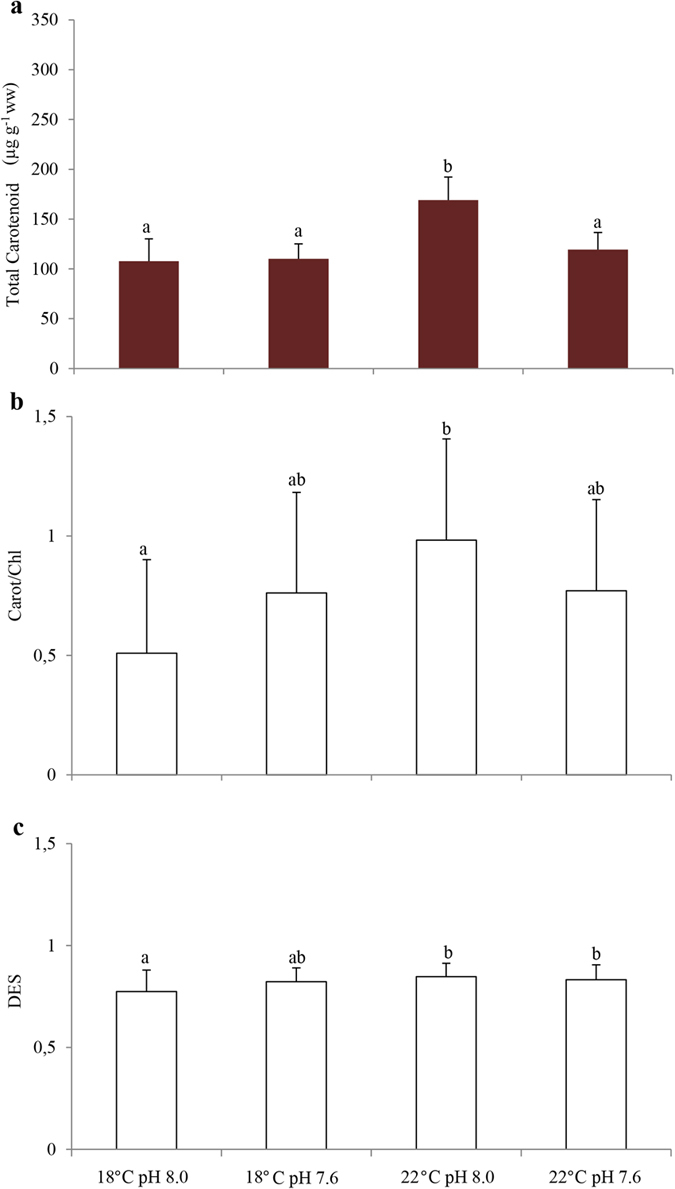
Impact of ocean warming (+4 °C) and acidification (ΔpH = 0.4) warming on *Zostera noltii*. (**a**) Total carotenoid content (μg g^−1^ ww); (**b**) carotene/chlorophyll ratio and (**c**) De-Epoxidation State (DES). Values represent mean ± SD [(n = 40 per experimental treatment (i.e., n = 10 per replicate system (n = 4), per experimental treatment)]. Different letters represent statistical differences between temperature and pH treatments, following a significant interaction (p < 0.013). Abbreviations: Carot/Chl: carotenoid/chlorophyll ratio; DES: De-epoxidation state; μg: microgram; g: gram; ww: wet weight.

Total carotenoid concentrations were significantly higher in seagrasses exposed to warming and normocapnia, in comparison to *Z. noltii* exposed to remaining scenarios (Fig. 5a; Supplementary Table S2). Additionally, total carotenoid concentration was positively correlated with: i) frequency of shoots with 100% brown leaves (r = 0.99; p < 0.05; Table 1); ii) ETR variability (r = 0.96; p < 0.05; Table 1); iii) antheraxanthin (r = 0.99; p < 0.05; Table 1) and iv) *β*-carotene (r = 0.97; p < 0.05; Table 1). While seagrasses exposed to warming scenario presented higher carotenoids/chlorophylls ratios, differences were only statistically significant when compared with those recorded for specimens under control conditions (Fig. 5b; Supplementary Table S2). Additionally, carotenoids/chlorophylls ratio was positively correlated with: i) ETR variability (r = 0.96; p < 0.05; Table 1); ii) pheophytin *b* (r = 0.97; p < 0.05; Table 1) and iii) lutein (r = 0.98; p < 0.05; Table 1). De-Epoxidation State (DES) showed significantly lower values in *Z. noltii* exposed to control conditions in comparison to warming scenarios (both under normocapnic and hypercapnic conditions, Fig. 5c; Supplementary Table S2). Moreover, DES was negatively correlated with: i) chlorophyll *a* (r = −0.96; p < 0.05; Table 1); ii) total chlorophylls (r = −0.95; p < 0.05; Table 1) and iii) total carotenoids (r = −0.95; p < 0.05; Table 1).

## Discussion

Seagrass ability to cope with increasing temperature is dependent upon thermal tolerance of each individual species and environmental conditions^25^. As temperature rises and thermal tolerance upper limits are exceeded, cellular and organism’s death will potentially occur^26^,^27^. In our study, increasing temperature (+4 °C above control conditions) was the most determinant stressor in *Z. noltii* survival and physiology, severely affecting shoot density, leaf coloration and profile of photosynthetic pigments. When comparing lower shoot density values within warming/hypercapnic scenario *versus* control conditions, a plausible explanation could derive from limited recovery capacity of seagrass to counterbalance increasing temperature^13^,^28^,^29^, enhanced by slow CO_2_ diffusion capacity into seagrass leaves^30^,^31^.

Increasing temperature has been recognized to promote deleterious effects to health and survival of seagrass species. Indeed, *Z. marina* shoot survival and production was found to be adversely affected by increasing temperature^32^. In another study, increasing temperature was found to enhance *Posidonia oceanica* shoot mortality events, even exceeding recruitment rates^13^. Likewise, a swift shoot mortality and leaf loss were observed for *Z. muelleri* exposed to an increase temperature (32 °C), merely 5 °C above this species optimal growth value^28^. Moreover, a mass mortality episode (>90%) was reported for *Amphibolis antartica,* after the occurrence of a heat wave event^15^. Although *Z. noltii* inhabits the intertidal zone and therefore, to a more or less extent, can be physiologically adapted to a fluctuating environment^33^, our findings clearly show that increasing temperature, can pose a major threat to this species ability to survive. Consequently, we argue that, under a continuous and prolonged exposure to ocean warming conditions, *Z. noltii* will be closer or even above its physiological performance thresholds.

It is generally accepted that ocean acidification will benefit photosynthesis and growth rates of primary producers. As aqueous carbon dioxide will tend to increase, it is expected that a physiological competiveness enhancement may occur in all those species that rely upon and are capable to promptly seize CO_2_^10^,^19^,^34^. Most marine seagrasses are considered to be under-saturated with respect to dissolved inorganic carbon^10^,^31^. The majority of seagrasses have been described as photosynthetic C3 plants, although some exceptions are in order, including *Z. noltii* C4 capability^35^,^36^ or facultative photosynthetic pathways under low CO_2_ levels^10^. Consequently, an increase in dissolved CO_2_ concentration could bring a beneficial output in terms of seagrass growth and photosynthetic machinery performance, regardless of their capacity to use CO_2_, HCO_3_^−^ or other inorganic carbon sources for photosynthesis related processes^10^,^18^. Although not completely in accordance with this theory, our results do not contradict these previous considerations. In fact, pH was not a determinant factor, but when taking into consideration some analyzed endpoints, *Z. noltii* was, to a certain extent, beneficiated. Our findings suggest that *Z. noltii* has the ability to partially overcome negative effects of ocean warming through the use of CO_2_, HCO_3_^−^ or both, as inorganic carbon sources to obtain energy for metabolic processes and consequently survive. Moreover, we argue that the present findings also indicate to a carbon-limitation status (under present day conditions), with increasing dissolved carbon availability unlocking arrested photochemical potential of plants^37^.

Seagrass stress level was also assessed by measuring photosystem II (PSII) photochemical efficiency through chlorophyll fluorescence emitted from plant leaves^38^. Maximum photochemical efficiency (F_v_/F_m_ ratio) provides a measurement of photosynthetic stress, i.e. gives an insight if PSII disturbance is caused by plant stress. Additionally, higher F_v_/F_m_ ratios (0.79–0.84) are considered optimal for the majority of plant species, indicating healthier plants^39^. *Zostera noltii* exposed to control conditions exhibited nearly optimal F_v_/F_m_ ratios, which can indicate a healthy photosynthetic condition. On the contrary, lowest values for F_v_/F_m_ ratio were observed in seagrasses exposed under warming conditions, thus clearly demonstrating a stress related condition as a result of increased temperature. Likewise, similar findings were previously reported regarding tropical seagrass species^40^.

In our study, increased CO_2_ levels did not significantly affected photochemical efficiency of *Z. noltii*. Indeed, several studies point out towards an enhancement of photobiological fitness of C3 plants (under increasing CO_2_ scenarios), in opposition to C4 plants^10^,^37^,^41^. Indeed, while C3 species appear to respond positively to increasing atmospheric CO_2_ levels, enhancing its light harvesting mechanisms and its photosynthetic efficiency, C4 plants appear to suffer from stress induced by rising CO_2_. In C3 plants, CO_2_ fertilization induces Rubisco full carboxylation capacity and photosynthetic enhancement, mostly due to an enhancement of ETR and lower quantities of dissipated energy. On the contrary, C4 plants are usually working at full potential, presenting little or even any photosynthetic enhancement under CO_2_ fertilization. Although carbon-harvesting mechanisms and incorporation seem to be improved by higher dissolved CO_2_ availability, the light-harvesting component of the photosynthetic apparatus show some signs of stress. Again, this confirms the above mentioned hypothesis of a typical response of a C4 species^37^,^42^,^43^. Nonetheless, our results also contradict, to a certain degree, previous studies where high ETR values and photosynthesis downregulation were reported (under CO_2_ enriched conditions), indicating an inorganic carbon photosynthesis limitation of *Z. noltii*, at present day conditions^44^. Thus, it seems that *Z. noltii* might retain both C3–C4 photosynthetic capacity, but further experimental work needs to be performed in order to sustain this hypothesis. In our study, *Z. noltii* also showed a decrease in ETR capacity under warming conditions. Thus, warming seems to directly impact the electron transport chain (ETC), probably by affecting the PSII donor side^45^.

Levels of photosynthetic related pigments (e.g. chlorophylls and carotenoids) can provide an insight on plant growth, photosynthetic and physiological status^46^. Under heat stress, PSII donor side is highly affected due to its lower thermostability^47^, thus reducing its ability to supply the ETC with electronic energy generated during light harvesting. This inevitably generates high amounts of excessive energy within the stroma that needs to be dissipated as to prevent PSII permanent damage^47^. In our study this seemed to be achieved through two pathways: i) energy quenching by auroxanthin and ii) xanthophyll cycle activity. *Zostera noltii* individuals exposed to warming showed a significant increase in auroxanthin content, a violaxanthin analogue^48^. Auroxanthin is a C5,8 epoxy carotenoid that has previously been shown to enhance aggregation-associated quenching in isolated LHC IIb^49^. The effectiveness of auroxanthin has been suggested to derive from the fact that its S1 energy level is higher than the one of violaxanthin^48^. This effect is explained by its structure, where the end groups of auroxanthin lie in the plane of the conjugated carbon double bond chain, as in zeaxanthin, whereas they are expected to be out-of-plane in violaxanthin. In fact, the C5,8 epoxide would hold the end group rigidly in-plane, explaining why it is even more effective than zeaxanthin and thus be an efficient energy quencher, under stress conditions^49^,^50^. The activation of energy dissipation pathway is typical from heat stress plants damaged at the PSII donor side^51^. The coupled activation of the xanthophyll cycle (increased DES) along with a higher auroxanthin synthesis suggests that warming-exposed individuals had higher needs to dissipate excessive energy than seagrass under control temperature treatments. It is worth noting that the increase in antioxidant pigments (e.g. *β*-carotene) in seagrass exposed to warming and acidification conditions, reinforces the need to counteract the effects of excessive redox potential accumulated inside its chloroplasts and sub consequent generation of reactive oxygen species (ROS). Another clear evidence of cellular stress was the production of chlorophyll degradation products (pheophytin *a* and pheophytin *b*), generated by the loss of the chlorophyll molecule, under stress conditions. This fact was clearly perceived in leaf coloration. Here, not only there was an elimination of chlorophyll molecules, but a concomitant increase in carotenoid content; this could point towards a stress situation in opposition to natural senescence where only chlorophyll is reabsorbed^52^. Once again this was not recorded for specimens in treatments with higher dissolved CO_2_ availability, yet again indicating an ameliorating effect, reduced stress levels and in this case by supplying the required carbon to dissipate the excessive energy within the cells^37^.

As biological catalyst substances, enzymes play an important role in chlorophyll biosynthesis. Once temperature directly affects enzyme activity, chlorophyll synthesis is expected to be affected^53^. Such trend was not observed in our study with temperature influence not being recorded in total chlorophyll content. However, our findings conflict with previous studies, that recorded a rise in chlorophyll concentration within *Ruppia drepanensis* leaves, with increasing temperature conditions^54^. These contradictory results could eventually be explained by species specific strategies by means of distinct acclimation capabilities^54^.

To the best of our knowledge, no previous studies have reported the combined effects of ocean warming and acidification on *Z. noltii* survival, photophysiology and photosynthetic output. Our results show that *Z. noltii* exhibited down-regulated acclimation potential to future projected warming conditions, with possible severe consequences at individual and subsequently ecosystem level^15^. Although this species inhabits unstable and fluctuating marine coastal environments, it seems that predictable climate change scenarios will pose major constraints to *Z. noltii* ability to cope with additional stressors, particularly ocean warming. Due to its key ecological role within coastal environments, seagrass long-term acclimation capacity and/or adaptation response to anthropogenic-derived stressors should be further investigated, in order to elucidate and predict species resilience towards predicted climate change scenarios^10^,^21^,^44^. Moreover, since seagrasses are important ecosystem builders and biological richness “enhancers” within coastal habitats, future research should address how marine biota (e.g. epiphytes, macroalgae and bacterial communities) that co-exist and biologically interact with marine angiosperms can be affected and influence seagrasses adaptive plasticity to climate change^55^,^56^.

## Methods

### Seagrass collection and acclimation

Dwarf seagrass *Zostera noltii* was collected with its natural sediment using a stainless steel core (300 × 200 × 100 mm, AISI 316), at a pristine area (38°29′ 18.42″N; 8°53′ 15.12″W, Caldeira de Tróia, Portugal)^57^. Following collection, each sample (seagrass and natural sediment; n = 16) was covered with seawater soaked towels (air dissection avoidance) within thermal insulated cases and immediately transported to the aquatic facilities of Laboratório Marítimo da Guia (FCUL, Portugal). Upon arrival, samples were laboratory acclimated, in order to minimize any changes in the natural sediment structure (collected with *Z. noltii*). Laboratory acclimation lasted 30 days, under seawater conditions mimicking those at collection site: salinity = 35 ± 1 (V2 refractometer, TMC Iberia, Portugal); water temperature = 18 ± 1 °C (TFX 430 Thermometer, WTW GmbH, Germany) and pH = 8.0 ± 0.1 (SG8 – SevenGo pro™ pH/Ion meter, Mettler-Toledo International Inc., Switzerland). After initial laboratory acclimation, seagrass was exposed to the following experimental conditions: i) control scenario (18 °C, pH 8.0); ii) hypercapnic scenario (18 °C, pH 7.6); iii) warming scenario (22 °C, pH 8.0) and iv) combined warming and hypercapnic scenario (22 °C, pH 7.6), following IPCC’s RCP scenario 8.5. Normocapnia [i.e. experimental conditions: i) and iii)] and hypercapnia [i.e. experimental conditions: ii) and iv)] conditions were defined as experimental settings where pH = 8.0 and 7.6, respectively. Experimental exposure lasted an additional 30 days and consisted in sixteen 10-L tank setups (4 independent replicate tanks per treatment; Supplementary Figure S1).

Flow-through aquatic systems were set (as described in Fig. 3d schematics)^58^, in order to maintain total alkalinity, dissolved inorganic carbon speciation due to bacterial activity and acidification of treatments. Experimental tanks were set in a completely randomized outline^58^. Natural seawater (NSW) was pumped from the sea into a seawater storage tank (5 m^3^ capacity). Subsequently, NSW was 0.35 μm filtered (Harmsco, Florida, USA) and UV-irradiated (Vecton 600, TMC Iberia, Portugal), before being supplied to mixing (n = 16) and experimental (n = 16) tanks. Overhead tank illumination was provided through artificial overhead lightning apparatus (Aquabeam 1500 Ultima NP, TMC Iberia, Portugal), with a correlated color temperature of 20.000 K, total luminous flux of 1965 lumens and a 120° beam angle. Photosynthetically active radiation (PAR) was measured using a portable fluorometer (FluorPen FP 100, Photo Systems Instruments, Czech Republic) and maintained at 180 ± 10 μmol photons m^−2^ s^−1^ (mid-light intensity for *Zostera* spp.)^59^. Experimental tanks were kept under a photoperiod of 14 h/10 h (light/dark cycle), according to prevailing natural light conditions (collection site). Ammonia and nitrite levels were daily checked using colorimetric tests (Salifert Profi Test, Holland), while individual pH values were adjusted automatically (Profilux 3.1, GHL, Germany). Monitoring of pH was performed every 2 seconds interval, with pH values being lowered through the injection of certified CO_2_ gas (Air Liquide, Portugal) or upregulated through aeration using CO_2_ filtered atmospheric air (soda lime, Sigma-Aldrich). Seawater temperature was controlled through seawater chilling systems (Frimar, Fernando Ribeiro Lda, Portugal) and submerged heaters (150 W, Eheim GmbH & Co. KG, Germany). Additionally, handheld equipment was used in order to perform a daily monitoring of seawater temperature (TFX 430 thermometer, WTW GmbH, Germany) and salinity (V2 refractometer, TMC Iberia, Portugal). Seawater carbonate system speciation was calculated weekly from total alkalinity (spectrophometrically at 595 nm) and pH measurements^60^. Seawater temperature and pH of different experimental setups is summarized in Supplementary Table S1. Quantification of pH was determined using a pH meter (826 pH mobile, Metrohm, Germany) connected to a glass electrode (Schott IoLine, SI analytics,±0.001), calibrated with TRIS-HCl (TRIS) and 2-aminopyridine-HCl (AMP) (Mare, Belgium) seawater buffers^61^. Recording of pH values was performed under temperature controlled conditions using a water bath (±0.1 °C, Lauda, Germany). Bicarbonate and *p*CO_2_ values (Supplementary Table S1) were calculated using the CO_2_SYS software (Carbon Dioxide Information Analysis Center, Oak Ridge National Laboratory, USA), with dissociation constants^62^, as refitted^63^.

### Shoot density and leaf color intensity

*Zostera noltii* shoot density was determined at the beginning of the experiment (T0) and 30 days (T30) after exposure to experimental conditions. Shoot density was recorded as the number of shoots per area (shoots.cm^−2^). Seagrass leaves color was assessed through direct observation (T30) and scored using the following criteria: i) 100% green; ii) 75% green and 25% brown; iii) 50% green and 50% brown and iv) 100% brown.

### Photobiology

Pulse amplitude modulation (PAM) fluorometry was used to monitor photosynthetic activity [40 leaves per treatment, 30 days (T30) after seagrass exposure to experimental conditions], by measuring non-intrusively variable chlorophyll fluorescence^64^. The PAM fluorometer comprised a computer-operated PAM-control unit (Junior-Pam, Heinz Walz GmbH, Germany) and a water-EDF-Universal emitter-detector unit (Gademann Instruments GmbH, Germany). Actinic and saturating light was provided by a blue LED-lamp (450 nm peak, 20 nm half-band width), supplied to the experimental biological sample by a fiber optic (fo) bundle (1.5 mm-diameter). The fo bundle was perpendicularly positioned to seagrass leaf surface. Measurements were taken 30 days after the beginning of experimental exposure. Seagrass replicates were dark-adapted for 30 min, after which one saturation pulse (0.8 s) was applied to determine the minimum- or dark-level fluorescence (*F*_o_), a parameter expected to correlate with chlorophyll *a* (chl *a*) content^65^ and the maximum fluorescence (*F*_m_). Photosystem II (PSII) quantum yield was determined using *F*_o_ and *F*_v_^64^ :


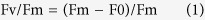


The F_v_/F_m_ ratio indicates the proportion of PSII reaction centers capable of converting captured light into photosynthetic energy (PSII quantum yield). This ratio is a convenient measure of the maximum potential quantum yield of PSII, and therefore is inversely proportional to photochemical stress.

Seagrasses were light-adapted for 30 minutes as to calculate the electron transport rate, a light-adapted parameter that is directly related to PS(II) by the equation:


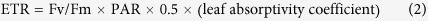


where 0.5 compensates for irradiance being split between two photosystems. The absorption coefficient used was 0.84.

### Gauss peak spectra pigment analysis

*Zostera noltii* leaves (n = 40 per treatment) were collected after 30 days of exposure to experimental conditions (T30) and immediately frozen. For pigment extraction, leaves were freeze-dried (48 h dark), after which were grinded (pure acetone, using a glass rod). To ensure complete disaggregation of leaf material, samples with acetone were subjected to a cold ultra-sound bath (2 min)^66^. Extraction occurred at −20 °C (24 h, dark) to prevent pigment degradation. After extraction, samples were centrifuged (4,000 rpm, 15 min at 4 °C). The Gauss-Peak Spectra method^67^ was employed for pigment analysis. This method has been widely applied for higher plants^40^,^47^ and proved to analyze pigment profile efficiently even when compared with HPLC analysis^67^. This method has the advantage of being less expensive and faster than HPLC analysis, providing a statistical analysis of the results while comparted to the peak shapes of standard materials. All analyses in which results did not match statistical standards were discarded, with extraction and subsequent analysis being repeated. Samples were scanned in a dual beam spectrophotometer (350 nm to 750 nm, 0.5 nm steps). Absorbance spectrum was introduced in the Gauss Peak Spectra (GPS) fitting library, thus allowing the identification and quantification of pigments (i.e. chlorophyll *a*, chlorophyll *b*, antheraxanthin, *β*-carotene, auroxanthin, lutein, violoxanthin and zeaxanthin), using SigmaPlot Software (Systat Software, Inc., USA). In order to better evaluate light harvesting and photo-protection mechanisms, De-Epoxidation State (DES) was calculated as:


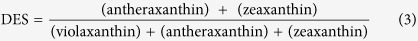


The xanthophyll cycle consists of light-dependent conversions of three xanthophylls in a cyclic reaction. Succinctly, it involves a deepoxidation sequence from the diepoxide violaxanthin, via the monoepoxide antheraxanthin to the epoxide-free form zeaxanthin, facilitating the de-excitation of accumulated chlorophyll singlet excited states^68^. This allows PS(II) to dissipate excessive energy in the form of heat. De-Epoxidation State evaluates the activity of this cyclic reaction, with higher values corresponding to higher conversions of the epoxides to zeaxanthin.

### Statistical analyses

In order to detect significant statistical differences in shoot density, a two-way ANOVA (temperature and pH as explaining factors) was performed. In addition, two separate two-way multivariate analysis of variance (MANOVA), using temperature and pH as explaining factors, were performed to identify significant effects of temperature and pH on: i) photophysiological parameters (ETR and F_v_/F_m_), and ii) pigment levels. As MANOVA revealed significant differences, two-way ANOVA followed by Tukey *post-hoc* tests were conducted (whenever the interaction between temperature and pH was significant) in order to better scrutinize the effect of explaining variables on each measured endpoint. Dunn-Sidak procedure was used to adjust the associated significance level of the family-wise type-I error. A total of 4 comparisons were applied (2 temperature levels combined with 2 pH values), resulting in a corrected significance level of 0.013. Prior to all analyses of variance, data were checked for normality and homoscedasticity (Kolmogorov-Smirnov and Levene’s tests, respectively).

A Pearson correlation analysis was also applied in order to investigate potential relationships between biological (shoot density, frequency of shoots’ coloration), photophysiological (ETR and F_v_/F_m_) and biochemical (pigments) variables. Unless stated otherwise, all statistical analyses were performed using a significance level of 0.05, using Statistica 10.0 software (StatSoft Inc., USA).

## Additional Information

**How to cite this article:** Repolho, T. *et al*. Seagrass ecophysiological performance under ocean warming and acidification. *Sci. Rep.* **7**, 41443; doi: 10.1038/srep41443 (2017).

**Publisher's note:** Springer Nature remains neutral with regard to jurisdictional claims in published maps and institutional affiliations.

## Supplementary Material

Supplementary Information

## Figures and Tables

**Table 1:** Pearson’s correlation coefficients between density, frequency of leaf colors, ETR, F_v_/F_m_ and pigments profile, with respective ratios and indices in the seagrass (*Zostera noltii*) under ocean warming and acidification.

	Dens	CF 100% Green	CF100% Brown	CF 75–15%	CF 50–50%	ETR	ETR Var	F_v_/F_m_	F_v_/F_m_Var	Chl *a*	Chl *b*	Total Chl	Pheo *a*	Pheo *b*	Auro	Anthe	*β* car	Lut	Viol	Zea	Total Car	Carot/Chl
Dens	1.00																					
CF - 100% G	0.49	1.00																				
CF- 100% B	−0.88	−0.11	1.00																			
CF - 75% G, 15%B	0.09	−0.13	−0.89	1.00																		
CF - 50% G, 50%B	0.14	−0.75	−0.56	0.66	1.00																	
ETR	0.91	0.80	−0.63	0.48	−0.28	1.00																
ETR Variance	−0.88	−0.30	**0.97**	−0.75	−0.39	−0.70	1.00															
F_v_/F_m_	0.84	0.81	−0.48	0.41	−0.40	**0.98**	−0.54	1.00														
F_v_/F_m_ Var	−0.94	−0.75	0.68	−0.55	0.21	−**0.99**	0.74	−**0.97**	1.00													
Chl *a*	0.68	0.85	−0.52	0.14	−0.34	0.82	−0.71	0.72	−0.80	1.00												
Chl *b*	0.51	**0.98**	−0.21	−0.12	−0.67	0.78	−0.42	0.76	−0.74	0.93	1.00											
Total Chl	0.65	0.86	−0.49	−0.11	−0.37	−0.81	−0.68	0.71	−0.78	**0.99**	0.94	1.00										
Pheo *a*	**−0.96**	−0.38	**0.96**	−0.80	−0.31	−0.81	**0.98**	−0.68	0.85	−0.70	−0.45	−0.68	1.00									
Pheo *b*	−0.90	−0.37	**0.96**	−0.73	−0.33	−0.75	**0.99**	−0.59	0.78	−0.74	−0.47	−0.72	**0.99**	1.00								
Auro	−0.92	−0.50	0.91	−0.67	−0.19	−0.83	**0.98**	−0.69	0.85	−0.82	−0.59	−0.80	**0.98**	**0.99**	1.00							
Anthe	−0.84	−0.05	**0.99**	−0.89	−0.61	−0.57	**0.96**	−0.41	0.62	−0.48	−0.15	−0.45	0.94	0.94	0.89	1.00						
*β* car	−0.80	0.09	**0.97**	**−0.96**	−0.70	−0.49	0.88	−0.36	0.55	−0.30	−0.03	−0.26	0.88	0.86	0.78	**0.98**	1.00					
Lut	−0.75	0.15	**0.96**	−0.93	−0.75	−0.42	0.88	−0.27	0.48	−0.28	−0.06	−0.25	0.86	0.85	0.77	**0.98**	**0.99**	1.00				
Viol	0.32	**0.97**	−0.00	−0.32	−0.79	−0.66	−0.23	0.65	−0.60	0.85	**0.98**	0.86	−0.26	−0.28	−0.42	0.05	0.23	0.26	1.00			
Zea	−0.64	0.32	0.90	−0.93	−0.85	−0.27	0.78	−0.13	0.34	−0.11	0.23	−0.08	0.75	0.75	0.65	0.93	**0.97**	**0.98**	0.43	1.00		
Total Car	−0.80	−0.03	**0.99**	−0.86	−0.63	−0.53	**0.96**	−0.36	0.58	−0.48	−0.14	−0.45	0.92	0.94	−0.80	**0.99**	**0.97**	−0.25	0.06	0.93	1.00	
Carot / Chl	−0.82	−0.51	0.85	−0.53	−0.16	−0.75	**0.96**	−0.59	0.77	−0.87	−0.63	−0.86	0.92	**0.97**	0.88	0.83	0.70	**0.98**	−0.48	0.58	−0.86	1.00
DES	−0.40	−0.72	0.73	−0.41	0.10	−0.87	0.87	−0.75	0.87	**−0.96**	−0.81	**−0.95**	0.87	0.90	0.95	0.70	0.55	0.53	−0.68	0.38	−**0.95**	**0.96**

Values in bold represent statistical significance at the 5% level of significance. Abbreviations: Dens – density; CF – leaf color frequency (G-Green; B- Brown); ETR – electron transport rate; ETR Var: electron transport rate variance; F_V_/F_m_: maximum PSII quantum yield; F_V_/F_m_ Var: maximum PSII quantum yield variance; Chl *a*: chlorophyll *a*; Chl *b*: chlorophyll *b*; Total Chl: total chlorophyll; Pheo *a*: pheophytin *a*; Pheo *b*: pheophytin *b*; Auro - Auroxanthin; Anthe: Antheraxanthin; β car: *β*-carotene; Lut: luteín; Viol: violaxanthin; Zea: zeaxanthin; Total Car: total carotenoid; Carot/Chl: carotenoid/chlorophyll ratio; DES: De-epoxidation state.
