# Supplementary material for: Seagrass ecophysiological performance under ocean warming and acidification
Source: Sci Rep. 2017 Feb 1;7:41443. doi: 10.1038/srep41443 (PMC5286439; doi:10.1038/srep41443)
Supplement: Supplementary Information [file srep41443-s1.doc]

**Supplementary Information**

**Seagrass ecophysiological performance under ocean warming and acidification**

Tiago Repolho ^1*^, Bernardo Duarte ^2^, Gisela Dionísio ^1,3^, José Ricardo Paula ^1^, Ana Lopes ^1^, Inês C. Rosa ^1^, Tiago Grilo ^1^, Isabel Caçador ^2^, Ricardo Calado ^3^, Rui Rosa ^1^

^1^ MARE - Marine and Environmental Sciences Centre, Laboratório Marítimo da Guia, Faculdade de Ciências da Universidade de Lisboa, Avenida Nossa Senhora do Cabo 939, 2750-374 Cascais, Portugal

^2^ MARE - Marine and Environmental Sciences Centre, Faculdade de Ciências da Universidade de Lisboa, Campo Grande, 1749‐016 Lisboa, Portugal

^3^ Departamento de Biologia & CESAM, Universidade de Aveiro, Campus Universitário de Santiago, 3810-193 Aveiro, Portugal.

^*^ Correspondence: Tiago Repolho, tel. +351 214869211, fax: +351 214869720, e-mail: [tfrepolho@fc.ul.pt](mailto:tfrepolho@fc.ul.pt)

Supplementary Figure 1 - Schematic representation of life support systems setup used in order to perform the experimental exposure of *Zostera noltii* to control, ocean warming and acidification conditions. Temperature (18 °C and 22 °C); normocapnia (pH 8.0); hypercapnia (pH 7.6).


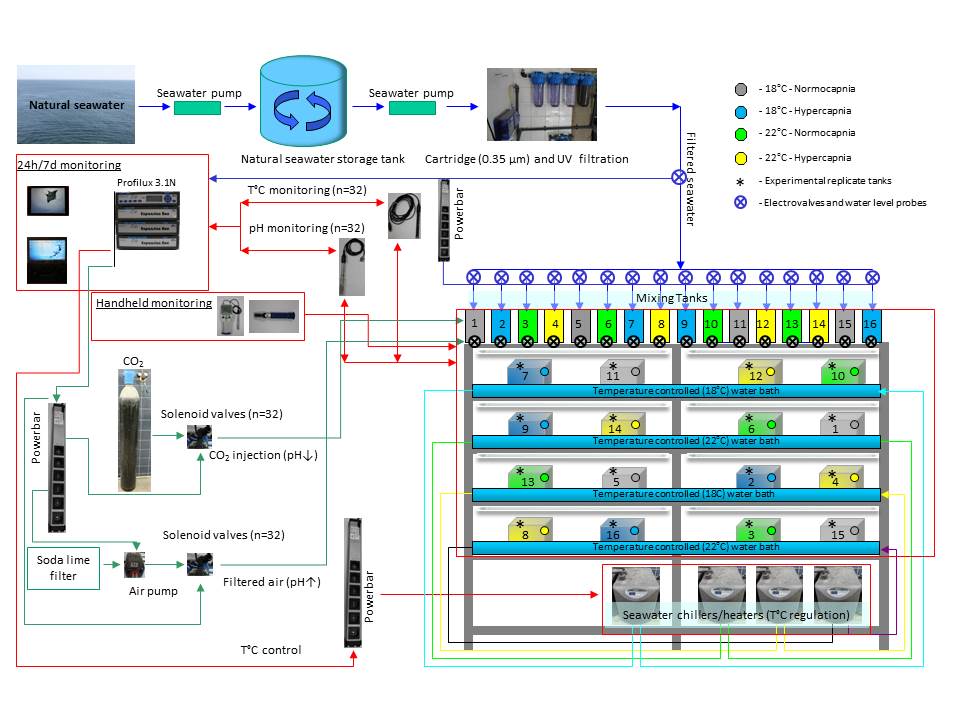


Supplementary Table 1 - Seawater physicochemical parameters (temperature and pH) in all experimental setups. Salinity and temperature were measured daily and averaged per replicate aquarium over the whole experimental period. The combination of total alkalinity (A_T_) and pH_T_ (pH total scale) was used to calculate carbonate system parameters [*p*CO_2_ (carbon dioxide partial pressure), C_T_ (total inorganic carbon) and Ω Arg (aragonite saturation state)]. Values are represented as mean ± standard deviation.

| Temperature | 18 °C | | | | | | |  | 22 °C | | | | | | |  |
| --- | --- | --- | --- | --- | --- | --- | --- | --- | --- | --- | --- | --- | --- | --- | --- | --- |
| pH | 8.0 | | |  | 7.6 | | |  | 8.0 | | |  | 7.6 | | |  |
| *measured* |  |  |  |  |  |  |  |  |  |  |  |  |  |  |  |  |
| Temperature (°C) | 18.0 | ± | 0.2 |  | 18.1 | ± | 0.2 |  | 21.9 | ± | 0.2 |  | 22.0 | ± | 0.2 |  |
| Salinity | 35.2 | ± | 0.9 |  | 34.5 | ± | 0.8 |  | 35.1 | ± | 1.1 |  | 35.4 | ± | 0.8 |  |
| pH_T_ | 8.00 | ± | 0.03 |  | 7.61 | ± | 0.03 |  | 8.01 | ± | 0.04 |  | 7.61 | ± | 0.02 |  |
| A_T_ (µmol kg^-1^ SW) | 1204.76 | ± | 105.04 |  | 1121.74 | ± | 104.55 |  | 1210.45 | ± | 105.61 |  | 1122.65 | ± | 99.03 |  |
|  |  |  |  |  |  |  |  |  |  |  |  |  |  |  |  |  |
| *calculated* |  |  |  |  |  |  |  |  |  |  |  |  |  |  |  |  |
| *p*CO_2_ (ppm) | 221.15 | ± | 3.74 |  | 580.42 | ± | 48.61 |  | 219.10 | ± | 4.90 |  | 595.01 | ± | 44.37 |  |
| C_T_ (µmol kg^-1^ SW) | 1051.64 | ± | 87.16 |  | 1071.18 | ± | 99.92 |  | 1036.53 | ± | 85.60 |  | 1062.25 | ± | 95.03 |  |
| Ω Arg | 1.29 | ± | 0.20 |  | 0.55 | ± | 0.08 |  | 1.50 | ± | 0.20 |  | 0.63 | ± | 0.07 |  |

Supplementary Table 2 – Summary of two-way ANOVA performed in order to assess the effects of temperature (T) and pH over *Zostera noltii* endpoints (i-vi), following a 30-day experimental exposure to conditions simulating present day and future climate change scenarios. i) shoot density; ii) photophysiological parameters [electron transport rate (ETR); maximum PSII quantum yield (F_V_/F_m_)]; iii) pigments; iv) total chlorophyll and total carotenoid; v) carotenoid/chlorophyll ratio and vi) De-epoxidation state (DES). Significant statistical values are marked in bold (see main manuscript for more details on α).

|  | **df** | **MS** | **F** | **p** |
| --- | --- | --- | --- | --- |
| **Shoot density** (α = 0.05) | | | | |
| *T* | 1 | 4556.25 | 63.83 | **< 0.001** |
| *pH* | 1 | 0.25 | 3.50x10^3^ | 0.954 |
| *T x pH* | 1 | 121.00 | 1.69 | 0.217 |
| *Error* | 12 | 71.37 |  |  |
| **Photophysiological parameters** (α = 0.013) | | | | |
| **ETR** |  |  |  |  |
| *T* | 1 | 2084.20 | 16.83 | **< 0.001** |
| *pH* | 1 | 412.60 | 3.33 | 0.070 |
| *T x pH* | 1 | 957.60 | 7.73 | **0.006** |
| *Error* | 182 | 123.90 |  |  |
| **F_V_/F_m_** |  |  |  |  |
| *T* | 1 | 0.57 | 41.59 | **< 0.001** |
| *pH* | 1 | 0.07 | 4.90 | 0.028 |
| *T x pH* | 1 | 1.00x10^-5^ | < 1.00x10^-5^ | 0.984 |
| *Error* | 153 | 0.01 |  |  |
| **Pigments** (α = 0.013) | | | | |
| **Chlorophyll *a*** | | | | |
| *T* | 1 | 24621.00 | 6.66 | **0.011** |
| *pH* | 1 | 19286.00 | 5.22 | 0.024 |
| *T x pH* | 1 | 21951.00 | 5.94 | 0.016 |
| *Error* | 103 | 3694.00 |  |  |
| **Chlorophyll *b*** | | | | |
| *T* | 1 | 1199.70 | 1.94 | 0.166 |
| *pH* | 1 | 3233.50 | 5.23 | 0.024 |
| *T x pH* | 1 | 555.90 | 0.90 | 0.344 |
| *Error* | 104 | 617.60 |  |  |
| **Total chlorophyll** |  |  |  |  |
| *T* | 1 | 35062.00 | 4.79 | 0.031 |
| *pH* | 1 | 32569.00 | 4.45 | 0.037 |
| *T x pH* | 1 | 34689.00 | 4.74 | 0.032 |
| *Error* | 103 | 7321.00 |  |  |
| **Pheophytin *a*** | | | | |
| *T* | 1 | 9559.33 | 26.00 | **< 0.001** |
| *pH* | 1 | 284.52 | 0.77 | 0.381 |
| *T x pH* | 1 | 2054.85 | 5.59 | 0.020 |
| *Error* | 99 | 367.69 |  |  |
| **Pheophytin *b*** |  |  |  |  |
| *T* | 1 | 2084.33 | 13.79 | **< 0.001** |
| *pH* | 1 | 68.47 | 0.45 | 0.502 |
| *T x pH* | 1 | 1008.07 | 6.67 | **0.011** |
| *Error* | 102 | 151.11 |  |  |
| **Auroxanthin** |  |  |  |  |
| *T* | 1 | 10809.20 | 16.88 | **< 0.001** |
| *pH* | 1 | 0.20 | 3.00x10^-4^ | 0.986 |
| *T x pH* | 1 | 4384.10 | 6.85 | **0.010** |
| *Error* | 102 | 640.20 |  |  |
| **Antheraxanthin** |  |  |  |  |
| *T* | 1 | 1297.06 | 25.06 | **< 0.001** |
| *pH* | 1 | 501.82 | 9.70 | **0.002** |
| *T x pH* | 1 | 499.61 | 9.65 | **0.002** |
| *Error* | 103 | 51.75 |  |  |
| ***β*-carotene** |  |  |  |  |
| *T* | 1 | 843.04 | 9.54 | **0.002** |
| *pH* | 1 | 564.80 | 6.39 | **0.013** |
| *T x pH* | 1 | 135.22 | 1.53 | 0.219 |
| *Error* | 105 | 88.39 |  |  |
| **Luteín** |  |  |  |  |
| *T* | 1 | 353.50 | 5.16 | 0.025 |
| *pH* | 1 | 323.31 | 4.72 | 0.032 |
| *T x pH* | 1 | 113.17 | 1.65 | 0.201 |
| *Error* | 104 | 68.43 |  |  |
| **Violaxanthin** |  |  |  |  |
| *T* | 1 | 25.34 | 1.15 | 0.286 |
| *pH* | 1 | 205.81 | 9.33 | **0.003** |
| *T x pH* | 1 | 19.60 | 0.89 | 0.348 |
| *Error* | 102 | 22.07 |  |  |
| **Zeaxanthin** |  |  |  |  |
| *T* | 1 | 363.70 | 3.71 | 0.057 |
| *pH* | 1 | 662.09 | 6.76 | **0.011** |
| *T x pH* | 1 | 109.10 | 1.11 | 0.294 |
| *Error* | 104 | 97.99 |  |  |
| **Total carotenoid** |  |  |  |  |
| *T* | 1 | 32097.00 | 13.45 | **< 0.001** |
| *pH* | 1 | 14599.00 | 6.12 | 0.015 |
| *T x pH* | 1 | 17497.00 | 7.33 | **0.008** |
| *Error* | 103 | 2386.00 |  |  |
| **Carotenoid/Chlorophyll ratio** | | |  |  |
| *T* | 1 | 1.50 | 9.20 | **0.003** |
| *pH* | 1 | 0.01 | 0.06 | 0.799 |
| *T x pH* | 1 | 1.39 | 8.53 | **0.004** |
| *Error* | 103 | 0.16 |  |  |
| **DES** |  |  |  |  |
| *T* | 1 | 0.046 | 7.03 | **0.009** |
| *pH* | 1 | 7.29x10^-3^ | 1.11 | 0.294 |
| *T x pH* | 1 | 0.03 | 4.00 | 0.048 |
| *Error* | 104 | 6.55x10^-3^ |  |  |
